# Microtransesophageal Echocardiographic Guidance during Percutaneous Interatrial Septal Closure without General Anaesthesia

**DOI:** 10.1155/2020/1462140

**Published:** 2020-09-07

**Authors:** Roel J. R. Snijder, Laura E. Renes, Martin J. Swaans, Maarten Jan Suttorp, Jurrien M. Ten Berg, Martijn C. Post

**Affiliations:** ^1^Department of Cardiology, St. Antonius Hospital, Nieuwegein, Netherlands; ^2^Department of Emergency Medicine, St. Antonius Hospital, Nieuwegein, Netherlands

## Abstract

**Objective:**

To study the safety and efficacy of microtransesophageal echocardiography (micro-TEE) and TEE during percutaneous atrial septal defect (ASD) and patent foramen ovale (PFO) closure.

**Background:**

TEE has proven to be safe during ASD and PFO closure under general anaesthesia. Micro-TEE makes it possible to perform these procedures under local anaesthesia. We are the first to describe the safety and efficacy of micro-TEE for percutaneous closure.

**Methods:**

All consecutive patients who underwent ASD and PFO closure between 2013 and 2018 were included. The periprocedural complications were registered. Residual shunts were diagnosed using transthoracic contrast echocardiography (TTCE). All data were compared between the use of TEE or micro-TEE within the ASD and PFO groups separately.

**Results:**

In total, 82 patients underwent ASD closure, 46 patients (49.1 ± 15.0 years) with TEE and 36 patients (47.8 ± 12.1 years) using micro-TEE guidance. Median device diameter was, respectively, 26 mm (range 10–40 mm) and 27 mm (range 10–35 mm). PFO closure was performed in 120 patients, 55 patients (48.6 ± 9.2 years, median device diameter 25 mm, range 23–35 mm) with TEE and 65 patients (mean age 51.0 ± 11.8 years, median device diameter 27 mm, range 23–35 mm) using micro-TEE. There were no major periprocedural complications, especially no device embolizations within all groups. Six months after closure, there was no significant difference in left-to-right shunt after ASD closure and no significant difference in right-to-left shunt after PFO closure using TEE or micro-TEE.

**Conclusion:**

Micro-TEE guidance without general anaesthesia during percutaneous ASD and PFO closure is as safe as TEE, without a significant difference in the residual shunt rate after closure.

## 1. Introduction

Transesophageal echocardiography (TEE) has proven to be safe and important during the guidance of transcatheter interventions for structural heart disease, such as closure of an atrial septal defect (ASD) or patent foramen ovale (PFO) [1–4]. One of the downsides of TEE is the relatively large probe that causes inconvenience for the patient, making general anaesthesia necessary. Another downside is the potential complications, such as local trauma to the oropharynx, esophagus, or stomach. Microprobe TEE (micro-TEE) has proven to be safe and effective during transcatheter cardiac interventions in neonates and infants since 2009 [5–7]. More recently, micro-TEE is safely used in adults as well [8, 9]. Using a significantly smaller probe makes general anaesthesia not necessary, which might result in fewer complications. The use of micro-TEE during ASD and PFO closure has not been reported earlier. We describe the safety and efficacy of micro-TEE in comparison with TEE for guidance of these procedures.

## 2. Methods

### 2.1. Population

All consecutive patients that underwent percutaneous ASD or PFO closure with micro-TEE guidance between January 2016 and November 2018 in the St. Antonius Hospital, Nieuwegein, the Netherlands, were included. These patients were compared to all consecutive patients that underwent percutaneous closure with TEE guidance between March 2013 and December 2015. All patients underwent TEE previous to the procedure during an outpatient clinic visit. The local ethics commission approved the study.

### 2.2. Probes

TEE guidance was performed using the X7-2t TEE probe (Philips Healthcare) with a shaft diameter of 10 mm and a head diameter of 16 × 12 × 40 mm. The imaging frequency ranges between 2 and 7 MHz.

**Figure 1:**
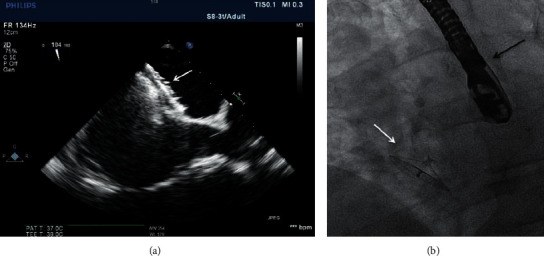
The Occlutech ASD device (white arrow) using the transesophageal echocardiography (TEE) probe (a) and fluoroscopy (b). The black arrow indicates the TEE probe during fluoroscopy.

**Figure 2:**
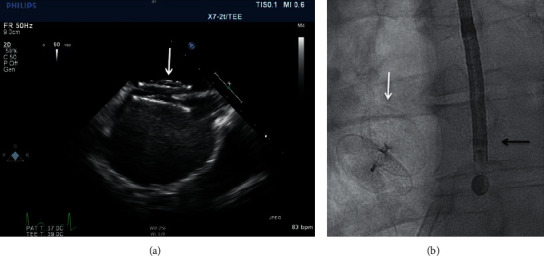
The Occlutech ASD device (white arrow) using the microtransesophageal echocardiography (micro-TEE) probe (a) and fluoroscopy (b). The black arrow indicates the micro-TEE probe during fluoroscopy.

The micro-TEE probe used was a S8-3t (Philips Healthcare) with a shaft diameter of 5.2 mm and a head diameter of 7.5 × 5.5 × 18.5 mm. The centre frequency is 6 MHz on a bandwidth of 3.2–7.4 MHz. Nijenhuis et al. described other specifications previously [8]. Both image modalities are presented in Figures 1 and 2.

### 2.3. Sedation Protocol

All patients received 2 to 6 sprays of Xylocaine (lidocaine 100 mg/ml) sprayed onto the oropharynx. When patients were anxious or restless during the procedures, diazepam 2.5–10 mg was administered intravenously. When light or moderate sedation was necessary, fentanyl (0.5–1 *μ*g/kg) or midazolam (0.025–0.05 mg/kg) was used, and propofol (0.5–0.75 mg/kg i.v.) was used for general anaesthesia.

### 2.4. Procedure

Percutaneous closure with TEE guidance was performed under general anaesthesia, and local anaesthesia was given in procedures where the micro-TEE was used. The interventional cardiologist chose the size of the device using balloon sizing and/or (micro-) TEE guidance. In all patients, the Occlutech Figulla Flex II device was used. The procedures are described in detail earlier [10, 11].

### 2.5. Follow-Up

All complications were registered. At 6-month follow-up, a contrast TTE (TTCE) with color Doppler was performed to determine the presence of a left-to-right shunt (LRS) in case of an ASD or for detecting a right-to-left shunt (RLS) after PFO closure using contrast. Successful ASD closure and PFO closure were defined as no residual LRS using color Doppler and no RLS or a minimal RLS, respectively. The RLS was graded as none, minimal, moderate, or severe [12].

### 2.6. Statistical Analysis

Descriptive statistics were used for patients' characteristics. Continuous variables with normal distribution are presented as mean ± standard deviation. All statistical analyses were performed using SPSS software (version 24.0 for Windows).

## 3. Results

### 3.1. Patient Characteristics

Between March 2013 and December 2015, percutaneous ASD closure was performed in 46 patients (mean age 49.1 ± 15.0 years, 52.5% female) and PFO closure in 55 patients (mean age 48.6 ± 9.2 years, 49.1% female) under TEE guidance.

Between January 2016 and November 2018, ASD closure was performed in 36 patients (mean age 47.8 ± 12.1 years, 52.8% female) and PFO closure in 65 patients (mean age 51.0 ± 11.8 years, 38.5% female) using micro-TEE guiding.

Within the ASD group, an atrial septal aneurysm (ASA) was present in 23.9% and 36.1% of the patients in both groups, respectively. Within both PFO groups, 40% of patients suffered an ASA.

Baseline characteristics and indication for closure are summarized in Table 1.

### 3.2. Procedural Outcome and In-Hospital Complications

All procedures using TEE guidance were performed under general anaesthesia, whereas all procedures using micro-TEE guidance were performed under local anaesthesia. Device implantation was successful in all patients, and no procedural complications occurred in all groups.

Within the ASD closure group, the median diameter of the device used was 26 mm (range 10–40 mm) in the TEE group and 27 mm (range 10–35 mm) in the micro-TEE group. PFO closure was performed using a device with a median diameter of 25 mm (range 23–35 mm) within the TEE group and 27 mm (23–35 mm) in the micro-TEE group.

After ASD closure, there was one patient (2.2%) with pericardial effusion without the need for intervention in the TEE group. New-onset supraventricular tachycardia (SVT) occurred in one patient (2.8%) in the micro-TEE group.

In-hospital, there was one patient (1.8%) after PFO closure pericardial effusion without the need for intervention in the TEE group and one patient (3.1%) with new-onset SVT in the micro-TEE group.

Periprocedural complications are summarized in Table 2.

### 3.3. Follow-Up

Six-month follow-up data were available in 72 patients (88%) after ASD closure (46 TEE and 26 micro-TEE) and in 99 patients (83%) after PFO closure (54 TEE and 45 micro-TEE). A TTE was available in 71 (87%) and 96 patients (80%) six months after ASD and PFO closure, respectively.

ASD closure was effective in 100% (no LRS) in the TEE group and in 92% in the micro-TEE group. PFO closure was effective (no or minimal RLS) in 51 patients (94%) in the TEE group and in 36 patients (86%) in the micro-TEE group. These differences were not statistically significant.

During follow-up, a stroke occurred in one patient after ASD closure in the TEE group. It concerned a 46-year-old man with a history of two transient ischemic attacks (TIAs) and a stroke with a recurrent stroke three weeks after closure; a thrombus on the device could not be diagnosed. The patient was still on dual platelet therapy and had a minimal RLS at 6-month follow-up and no RLS at 12-month follow-up.

## 4. Discussion

Micro-TEE guidance during interatrial septal defect closure under local anaesthesia is safe, but shows a nonsignificant difference in efficacy during six-month follow-up when compared to standard TEE and general anaesthesia.

### 4.1. Procedural Complications

In the literature, TEE guiding during percutaneous cardiac interventions has proven to be safe and effective [1–4]. Also, several studies have shown that micro-TEE is safe in infants and adults during percutaneous cardiac interventions [5–9]. None of these studies show periprocedural complications related to the use of TEE or micro-TEE. Complications that could occur using (micro-) TEE are trauma to the oropharynx, esophagus, or stomach. The advantage of local anaesthesia is spontaneous respiration, with a reduced risk for patients with cardiac and pulmonary comorbidities. There is also less need for nursing and monitoring with lower costs compared to general anaesthesia. However, local anaesthesia has disadvantages as well, such as a higher risk of anxiety, stress, and discomfort during the procedure. Ehret et al. compared general and local anaesthesia in a systematic review and meta-analysis in patients undergoing transcatheter aortic valve implantation [13]. They found that both types of anaesthesia are equally safe. There was no difference in in-hospital and 30-day mortality and other complications. In our study, there were no TEE- or micro-TEE-related complications as well. Furthermore, there were no complications due to general or local anaesthesia. The hospitalization duration was equal in both groups. Importantly, in none of the subgroups, a device embolization occurred.

### 4.2. Image Quality

The difference in image quality was previously published by Border et al. They studied the difference in image quality between TEE, micro-TEE, and intracardiac echocardiography (ICE) during left atrial appendage closure and MitraClip implantation. Image quality was comparable between micro-TEE and ICE, and the accuracy of micro-TEE is comparable to TEE. An important difference between TEE and micro-TEE is that micro-TEE has no three-dimensional view possibilities, which could help in sizing the defect and positioning the device [8]. Klett et al. compared micro-TEE and TEE as well during different types of interventions (transfemoral aortic valve implantation, transfemoral mitral valve implantation, and ASD and PFO closure). They found that micro-TEE provides good anatomical image quality of relevant structures during these interventions [9].

### 4.3. Residual Shunts

A residual LRS after ASD closure varies between 0% and 10% during a follow-up period between 6 months and 5 years [14–17]. In our study, a residual LRS was present in 24% in the TEE group and in 8.3% in the micro-TEE group immediately after ASD closure. During 6-month follow-up, the LRS resolves completely in the TEE group (0%) but was unchanged in the micro-TEE group (8.0%) using color Doppler echocardiography. The difference between these groups was not significant (*P*=0.063). Unfortunately, the percentages of patients who underwent a TTE at 6-month follow-up differed significantly between both groups (100% versus 69%). The difference between in-hospital and 6-month follow-up is unclear. It is possible that a better device position thanks to better image quality may cause an optimized device endothelialization.

After PFO closure, the residual RLS rate ranges from 3% to 39% during a follow-up period between 6 months and 2 years [18–21]. The large difference between the studies can be explained by the difference in the population size, but more importantly, by the difference in the definition and method used for the diagnosis of RLS. In our study, a moderate-to-severe RLS was present in 5.6% in the TEE group and in 14.3% in the micro-TEE group (*P*=0.197) at 6-month follow-up. Our population was relatively small, which could explain why the difference was not significant. The residual shunt rate of the micro-TEE group increased during 6-month follow-up when compared to in-hospital. It is possible that the patients that were lost to follow-up had no RLS or minimal RLS at follow-up creating a false high RLS rate.

It is unclear why this difference occurred. In the literature, defect size, presence of an ASA, type and size of the device size, and experience of the operator influence the chance of a residual shunt after percutaneous ASD and PFO closure [22, 23]. Firstly, balloon sizing for measuring the defect size was used in all patients in the ASD group making under- and oversizing unlikely. As balloon sizing was not used in percutaneous PFO closure, choosing the device size was based on imaging during the procedure. In both the ASD and PFO groups, there was no difference in defect size in the subgroups. Secondly, the defect anatomy was determined by TEE previous to the procedure in all patients. The presence of an ASA was equal in all groups making this an unlikely cause. Thirdly, there was no significant difference in device size between all groups. Fourthly, there was no difference in device choice as all defects were closed using the same device. Finally, the operators were very experienced and performed all procedures. There were no differences between operators.

As the predictors mentioned above did not show any difference between both groups, the only difference was the image modality. Because device position and device size (during PFO closure) were chosen using TEE and micro-TEE, the cause of the differences could be the image quality between these modalities. It is possible that the difference in image quality might cause undersizing or incorrect positioning of the device. However, previous studies did not find a difference in image quality during cardiac procedures [8, 9]. More studies are needed to show if there is an actual difference. Further, there was an important difference in available follow-up data making it more difficult to compare.

### 4.4. Arrhythmias and Cerebrovascular Events

A common complication after percutaneous ASD and PFO closure is new-onset SVTs. New-onset SVTs can occur in up to 3.5% of the patients after ASD closure [16, 17, 24] and can rise up to 6.6% after PFO closure as described in large trials [24–26].

Arrhythmias after ASD or PFO closure can occur due to oversizing of the device causing stress on the atrial cells of the interatrial septum. Therefore, the larger the device, the more chance on the development of new-onset SVTs [27].

In our study, there was no significant difference in new-onset SVTs in both the ASD and PFO groups, in-hospital and at 6-month follow-up.

After ASD closure and PFO closure, a recurrent cerebrovascular event has been described in, respectively, 0% to 0.3% and 0% to 5% [11, 15–18, 24, 28]. In our study, there was a recurrent stroke in one patient who underwent ASD closure (TEE group). After PFO closure, there were no recurrent cerebral ischemic events in both groups during 6-month follow-up. Though the complication rate is low, our follow-up time is relatively short.

### 4.5. Costs

Besides improving the comfort of the patient, local anaesthesia makes general anaesthesia (including anaesthesiology backup) unnecessary during this procedure and therefore reduces costs.

Ahmad et al. did a cost analysis of patients receiving a transcatheter aortic valve replacement with general anaesthesia versus conscious sedation [29]. Though hospitalization was shorter in the conscious sedation group, the total costs were not significantly different. Further, studies describing the costs of different surgical interventions under local versus general anaesthesia found a significant reduction in cost when performing the intervention under local anaesthesia [30, 31].

In our cohort, we studied local versus general anaesthesia instead of conscious sedation, making it difficult to compare the study of Ahmad et al. As the other studies did compare local versus general anaesthesia, we can conclude that using local anaesthesia can lower the costs of the procedure.

### 4.6. Limitations

Firstly, it was a single centre, nonrandomized study. Secondly, our study had a relatively small population making it more difficult to compare both image modalities. We used (contrast) TTE at follow-up for detecting a residual shunt while TEE is the gold standard. Finally, because 15% of the micro-TEE patients were lost to follow-up, it is possible that the event rate and residual shunting rate might differ significantly.

## 5. Conclusion

Using the micro-TEE during percutaneous ASD and PFO closure is safe when compared to standard TEE, but it seems to be associated with a nonsignificant increase in the shunt rate. However, it is less invasive without the need for general anaesthesia and no periprocedural complications.

## Figures and Tables

**Table 1:** Baseline characteristics.

	ASD			PFO		
	TEE	Micro-TEE	*P* value	TEE	Micro-TEE	*P* value
Number	46	36		55	65	
Age (years)^*∗*^	49.1 ± 15.0	47.8 ± 12.1	0.682	48.6 ± 9.2	51.0 ± 11.8	0.227
Female, *n* (%)	53 (52.5%)	19 (52.8%)	0.735	27 (49.1%)	25 (38.5%)	0.242
BMI (kg/m^2^)^*∗*^	27.2 ± 5.9	26.9 ± 4.9	0.733	24.9 ± 3.1	25.5 ± 4.3	0.520
*Risk factors and comorbidities, n (%)*						
Smoking	7 (15.2%)	3 (13.9%)	0.866	11 (20%)	6 (9.2%)	0.092
Diabetes	4 (8.7%)	2 (5.6%)	0.588	2 (3.6%)	4 (6.2%)	0.528
Arterial hypertension	13 (28.3%)	6 (16.7%)	0.222	14 (45.5%)	9 (13.8%)	0.107
Hypercholesterolemia	10 (21.7%)	5 (13.9%)	0.358	20 (36.4%)	10 (15.4%)	0.010
CAD	0 (0.0%)	1 (2.8%)	0.255	0 (0%)	2 (3.1%)	0.190
History of SVT	7 (15.2%)	1 (2.8%)	0.060	1 (1.8%)	2 (3.1%)	0.660
Positive family history	9 (19.6%)	6 (16.7%)	0.736	15 (27.3%)	12 (18.5%)	0.249
*TEE characteristics, n (%)*						
RLS after Valsalva	—	—		55 (100%)	65 (100%)	
Floppy/ASA	11 (23.9%)	13 (36.1%)	0.328	22 (40.0%)	26 (40.0%)	1.000
LRS	46 (100%)	36 (100%)		—	—	
ASD diameter (mm) ‡	15 (11–34)	15 (9–27)		—	—	

^*∗*^Data are presented as mean ± SD or number (percentage). ‡ Data are presented as median (range). ASD, atrial septal defect; PFO, patent foramen ovale; TEE, transesophageal echocardiography; BMI, body mass index; CAD, coronary artery disease; SVT, supraventricular arrhythmia; RLS, right-to-left shunt; ASA, atrial septal aneurysm; LRS, left-to-right shunt.

**Table 2:** In-hospital follow-up data after percutaneous ASD and PFO closure using TEE and micro-TEE.

	ASD		PFO	
	TEE	Micro-TEE	TEE	Micro-TEE
Number, *n*	46	36	55	65
Device diameter (mm)^*∗*^	26 (10–40)	27 (10–35)	25 (23–35)	27 (23–35)
*Complications, n (%)*				
SVT	0 (0%)	1 (2.8%)	0 (0%)	2 (3.1%)
Device embolism	0 (0%)	0 (0%)	0 (0%)	0 (0%)
Bleeding no transfusion	0 (0%)	0 (0%)	0 (0%)	0 (0%)
Pericardial effusion	1 (2.2%)	0 (0%)	1 (1.8%)	0 (0%)
*TTE, n*	46 (100%)	36 (100%)	55 (100%)	65 (100%)
*RLS, n (%)*				
No shunt	—	—	20 (36.4%)	53 (81.5%)
Minimal	—	—	26 (47.3%)	8 (12.3%)
Moderate	—	—	4 (7.3%)	2 (3.1%)
Severe	—	—	5 (9.1%)	2 (3.1%)
*LRS, n (%)*	11 (24%)	3 (8.3%)	—	—

^*∗*^Data are presented as median (range); ASD, atrial septal defect; PFO, patent foramen ovale; TEE, transesophageal echocardiography; SVT, supraventricular tachycardia; TTE, transthoracic echocardiography; RLS, right-to-left shunt; LRS, left-to-right shunt.

## Data Availability

The study data used to support the findings of this study are restricted by the Ethical Board of the St. Antonius Hospital in order to protect patient privacy. Data are available from the corresponding author, St. Antonius Hospital, for researchers who meet the criteria for access to confidential data.

## References

[1] Alqahtani F., Bhirud A., Aljohani S. (2017). Intracardiac versus transesophageal echocardiography to guide transcatheter closure of interatrial communications: nationwide trend and comparative analysis. *Journal of Interventional Cardiology*.

[2] Xu W., Li J., Ye J., Yu J., Yu J. G., Zhang Z. (2018). Transesophageal echocardiography and fluoroscopy for percutaneous closure of atrial septal defects: a comparative study. *Medicine (Baltimore)*.

[3] Scacciatella P., Meynet I., Giorgi M. (2018). Angiography vs. transesophageal echocardiography-guided patent foramen ovale closure: a propensity score matched analysis of a two-center registry. *Echocardiography*.

[4] Wunderlich N. C., Beigel R., Swaans M. J., Ho S. Y., Siegel R. J. (2015). Percutaneous interventions for left atrial appendage exclusion. *JACC: Cardiovascular Imaging*.

[5] Ho H. F., Chelliah A., Sable C. A. (2014). Evaluation of a second-generation microtransesophageal echocardiography transducer and software. *World Journal for Pediatric and Congenital Heart Surgery*.

[6] Sivakumar K., Arke A. D., Pavithran S., Natarajan K., Vishwambara B. (2014). Preliminary evaluation of a microtransesophageal probe in neonates and young infants undergoing surgery for congenital heart disease. *Annals of Pediatric Cardiology*.

[7] Toole B. J., Slesnick T. C., Kreeger J. (2015). The miniaturized multiplane micro-transesophageal echocardiographic probe: a comparative evaluation of its accuracy and image quality. *Journal of the American Society of Echocardiography*.

[8] Border V. J., Alipour A., Wunderlich N. C. (2017). Feasibility of multiplane microtransoesophageal echocardiographic guidance in structural heart disease transcatheter interventions in adults. *Netherlands Heart Journal*.

[9] Klettas D., Alcock E., Dworakowski R., MacCarthy P., Monaghan M. (2017). Is transnasal TEE imaging a viable alternative to conventional TEE during structural cardiac interventions to avoid general anaesthesia? A pilot comparison study of image quality. *Echo Research and Practice*.

[10] Van Den Branden B. J. L., Post M. C., Plokker H. W. M., Ten Berg J. M., Suttorp M. J. (2011). Percutaneous atrial shunt closure using the novel Occlutech Figulla device: 6-month efficacy and safety. *Journal of Interventional Cardiology*.

[11] Ten Berg R. J. R., Renes L. E., Suttorp M. J., Ten Berg J. M., Post M. C. (2019). Percutaneous patent foramen ovale closure using the Occlutech Figulla device: more than 1,300 patient-years of follow up. *Catheterization and Cardiovascular Interventions*.

[12] Van Gent M. W. F., Post M. C., Snijder R. J. (2009). Grading of pulmonary right-to-left shunt with transthoracic contrast echocardiography. *Chest*.

[13] Ehret C., Rossaint R., Foldenauer A. C. (2017). Is local anaesthesia a favourable approach for transcatheter aortic valve implantation? A systematic review and meta-analysis comparing local and general anaesthesia. *BMJ Open*.

[14] Pac A., Polat T. B., Cetin I., Oflaz M. B., Balli S. (2009). Figulla ASD Occluder versus Amplatzer Septal Occluder: a comparative study on validation of a novel device for percutaneous closure of atrial septal defects. *Journal of Interventional Cardiology*.

[15] Oflaz F., Houeijeh A., Recher M. (2015). Transcatheter closure of atrial septal defect with the Figulla ASD Occluder: a comparative study with the Amplatzer Septal Occluder. *Archives of Cardiovascular Diseases*.

[16] Pedra C. A. C., Pedra S. F., Costa R. N. (2016). Mid-term outcomes after percutaneous closure of the secundum atrial septal defect with the figulla-occlutech device. *Journal of Interventional Cardiology*.

[17] Haas N. A., Soetemann D. B., Ates I. (2006). Closure of secundum atrial septal defects by using the Occlutech Occluder devices in more than 1300 patients: the IRFACODE project: a retrospective case series. *Catheterization and Cardiovascular Interventions*.

[18] Saguner A. M., Wahl A., Praz F. (2011). Figulla PFO Occluder versus Amplatzer PFO Occluder for percutaneous closure of patent foramen ovale. *Catheterization and Cardiovascular Interventions*.

[19] Braun M., Gliech V., Boscheri A. (2004). Transcatheter closure of patent foramen ovale (PFO) in patients with paradoxical embolism Periprocedural safety and mid-term follow-up results of three different device occluder systems. *European Heart Journal*.

[20] Wahl A., Meier B., Schwerzmann M. (2005). Transcatheter treatment of atrial septal aneurysm associated with patent foramen ovale for prevention of recurrent paradoxical embolism in high-risk patients. *Journal of the American College of Cardiology*.

[21] Hildick-Smith D., Williams T., MacCarthy P. (2017). Occlutech percutaneous patent foramen ovale closure: safety and efficacy registry (OPPOSE). *International Journal of Cardiology*.

[22] Fleming R. G., Kumar P., West B. (2020). Comparison of residual shunt rate and complications across 6 different closure devices for patent foramen ovale. *Catheterization and Cardiovascular Interventions*.

[23] Marchese N., Pacilli M. A., Inchingolo V., Fanellei R., Loperfido F., Vigna C. (2013). Residual shunt after percutaneous closure of patent foramen ovale with AMPLATZER occluder devices - influence of anatomic features: a transcranial Doppler and intracardiac echocardiography study. *EuroIntervention*.

[24] Aytemir K., Oto A., Özkutlu S. (2013). Transcatheter interatrial septal defect closure in a large cohort: midterm follow-up results. *Congenital Heart Disease*.

[25] Mas J.-L., Derumeaux G., Guillon B. (2017). Patent foramen ovale closure or anticoagulation vs. Antiplatelets after stroke. *New England Journal of Medicine*.

[26] Søndergaard L., Kasner S. E., Rhodes J. F. (2017). Patient foramen ovale closure or antiplatelet therapy for cryptogenic stroke. *New England Journal of Medicine*.

[27] Komar M., Przewłocki T., Olszowska M. (2014). Conduction abnormality and arrhythmia after transcatheter closure of atrial septal defect. *Circulation Journal*.

[28] Aytemir K., Oto A., Özkutlu S. (2012). Early-mid term follow-up results of percutaneous closure of the interatrial septal defects with Occlutech Figulla devices: a single center experience. *Journal of Interventional Cardiology*.

[29] Ahmad M., Patel J. N., Vipparthy S. C. (2019). Consious sedation versus general anesthesia in transcatheter aortic valve replacement: a cost and outcome analysis. *Cureus*.

[30] Chandran D., Woods C. M., Schar M., Ma N., Ooi E. H., Athanasiadis T. (2018). Cost analysis of injection laryngoplasty performed under local anaesthesia versus general anaesthesia: an Australian perspective. *The Journal of Laryngology & Otology*.

[31] Locke M. C., Davis J. C., Brothers R. J., Love W. E. (2018). Assessing the outcomes, risks, and costs of local versus general anesthesia: a review with implications for cutaneous surgery. *Journal of the American Academy of Dermatology*.

